# Autonomous apomixis in *Praxelis clematidea* (Asteraceae: Eupatorieae), an invasive alien plant

**DOI:** 10.1093/aobpla/plab007

**Published:** 2021-01-29

**Authors:** Yuhuan Zhang, Hairong Wu, Elvira Hörandl, Rafael de Oliveira Franca, LiXin Wang, Jianhua Hao

**Affiliations:** 1 School of Biology and Food Engineering, Changshu Institute of Technology, Nansanhuanlu 99, Changshu 215500, Jiangsu Province, China; 2 College of Life Sciences, Northwest Normal University, No. 967, Anningxi Road, Lanzhou City 730070, Gansu Province, China; 3 Guangzhou Customs Technology Center, No. 66, Huacheng Avenue, Guangzhou 51062, Guangdong Province, China; 4 Department of Systematics, Biodiversity and Evolution of Plants (with Herbarium), University of Goettingen, Untere Karspuele 2, 37073 Goettingen, Germany; 5 Programa de Pós-graduação em Biologia Comparada, Universidade Estadual de Maringá, 87020-900 Maringá, Paraná, Brazil

**Keywords:** *Antennaria*-type, Apomixis, autonomous seed production, diplospory, embryological development, flow cytometric seed screening, invasive alien plant, pollination experiment, *Praxelis clematidea*

## Abstract

Understanding the reproductive mechanisms of invasive alien species can lay the foundation for effective control measures. *Praxelis clematidea* is a triploid neotropical Asteraceae species that is invasive in China and other countries. However, few studies have focused on its reproductive biology. In this study, flow cytometric seed screening (FCSS) was used to identify and confirm the reproductive mode of the species. The development of ovules, anthers, and mega- and microgametophytes was observed using a clearing method and differential interference contrast microscopy. Pollen viability was measured using the Benzidine test and Alexander’s stain. Pollen morphology was observed via fluorescence microscopy after sectioning the disk florets and staining with water-soluble aniline blue or 4′6-diamidino-2-phenylindole nuclei dyes. Controlled pollination experiments were conducted on four populations in China to examine the breeding system and to confirm autonomous apomixis. The reproductive mode was found to be autonomous apomixis without pseudogamy, according to FCSS. Megaspore mother cells developed directly into eight-nucleate megagametophytes without meiosis, conforming to *Antennaria*-type diplospory. The unreduced egg cells developed into embryos through parthenogenesis, while the endosperm was formed by the fusion of two unreduced polar nuclei. Pollen viability was very low (0.82 ± 0.57 % and 0.36 ± 0.44 %) as measured by the Benzidine test and Alexander’s stain, respectively. The majority of the pollen grains were empty and had neither cytoplasm nor nuclei. The seed set was >90 % for all treatments of open pollination, bagging and emasculated capitula. Mature cypselae developed in capitula that were emasculated before flowering, which confirmed that the breeding system of *P. clematidea* was autonomous apomixis. The present study is the first report of autonomous apomixis in *P. clematidea* in China. *Antennaria*-type autonomous apomixis in *P. clematidea* greatly increases the probability of successful colonisation and dispersal of *P. clematidea* into new areas, which likely contributes to its high invasion potential. Effective control measures should be implemented to prevent autonomous (pollen-independent) seed production.

## Teaser

*Praxelis* (*Praxelis clematidea*) belongs to Asteraceae like sunflower and originated in South America. It has become invasive in China and other countries. Its mode of reproduction was previously unknown. A team from China, Germany and Brazil explored the process of embryo sac and seed formation of *Praxelis*. They found that the seeds of *Praxelis* can develop by apomixis, a process of asexual seed formation. The plant embryo develops from an unreduced egg cell, and seed formation does not depend on fertilization with sperm, like in the majority of sexually reproducing plants. This special way of pollen-independent seed production is called autonomous apomixis and likely fosters the spread of invasive plants.

## Introduction

Biological invasion refers to the rapid spread and establishment of populations in non-origin ranges after long-distance dispersal (Barrett *et al.* 2008). Invasive alien plant species are one of the greatest threats to the sustainable development of global ecosystems and biodiversity (Perrings *et al.* 2010), and they have been recognized worldwide as one of the most serious ecological and economic threats since the new millennium (Pimentel 2002).

According to Baker’s law, self-propagating or apomictic species can more easily establish populations after long-distance dispersal than outcrossers that rely on conspecific male gametes from another individual and pollinators to reproduce (Baker 1967). Studies have shown that plants with the capacity for autonomous seed production are more likely to naturalize in introduced areas and become invasive than those with self-incompatibility systems or those that rely on pollinators (Rambuda *et al.* 2004; van Kleunen *et al.* 2008; Hao *et al.* 2011; Razanajatovo *et al.* 2016).

Two types of breeding systems lead to autonomous seed production: autogamy (selfing) and apomixis. Autogamy refers to the successful fertilization of female gametes by male gametes from the same flower after self-pollination (Cardoso *et al.* 2018). In these cases, pollination is carried out within flowers, and seeds can be produced even without pollinators. For the Asteraceae family, selfing means that seeds can be produced within a capitulum because the Asteraceae capitulum is thought to be a single large inflorescence and is a unique pollination unit (Burtt 1961). Apomixis is defined as a natural process that allows clonal reproduction through seeds, avoiding meiosis and fertilization via parthenogenetic development of an unreduced egg or a somatic cell (Nogler 1984; Koltunow and Grossniklaus 2003; Bicknell and Koltunow 2004) and is synonymous with asexual seed formation or agamospermy (Asker and Jerling 1992). Apomixis in angiosperms can be subdivided into two basic types: gametophytic and sporophytic apomixis. Gametophytic apomixis involves the formation of an unreduced female gametophyte (equal to the embryo sac or megagametophyte) within the ovule and the parthenogenetic development of the egg cell, whereas sporophytic apomixis involves the development of an embryo directly from a somatic cell within the ovule (Nogler 1984; Asker and Jerling 1992; Koltunow and Grossniklaus 2003; Bicknell and Koltunow 2004; Hu 2005; Hao and Qiang 2009; Bicknell and Catanach 2015). Gametophytic apomixis can utilize both diplospory and apospory as alternative pathways to form unreduced embryo sacs. In diplospory, the meiosis of megaspore mother cells (MMCs) is circumvented or restitutional, and a megagametophyte with maternal ploidy is produced. In apospory, unreduced megagametophytes arise from the somatic cell(s) of the nucellus (so-called aposporous initials). In both cases, mature embryos with maternal ploidy are formed (Nogler 1984; Asker and Jerling 1992; Koltunow and Grossniklaus 2003; Bicknell and Koltunow 2004; Hu 2005; Hao and Qiang 2009; Bicknell and Catanach 2015). When embryos and endosperm develop independently of fertilization, it is known as autonomous apomixis (Nogler 1984; Asker and Jerling 1992; Cardoso *et al.* 2018).

It is non-trivial to screen apomicts and estimate the incidence level of apomixis in a particular species or genotype. However, confirming the existence of apomictic events and estimating their occurrence are often arduous processes (Meier *et al.* 2011). Flow cytometric seed screening (FCSS) and ovule clearing combined with differential interference contrast (DIC) microscopy have made it possible to investigate large volumes of plant parts, including seeds, in the early stages of their development (Herr 1971, 1974; Young *et al.* 1979; Matzk *et al.* 2000; Krahulcová and Suda 2006; Hao and Qiang 2007; Hao and Shen 2009; Hojsgaard *et al.* 2014a). According to the principle of FCSS, the breeding system of plants can be inferred from the ploidy levels of the embryo and endosperm within the mature dry seeds (Matzk *et al.* 2000). Sexual seeds develop the embryo from a reduced egg cell fertilized by a reduced sperm cell (n + n), and the endosperm from two reduced polar nuclei fertilized by a sperm nucleus (2n + n); hence, a 2:3 ratio of embryo:endosperm peaks is observed in the flow cytometric histograms. Seeds formed via autonomous apomixis have an embryo from an unreduced egg cell (2n) and an endosperm from two unreduced polar nuclei (4n), both unfertilized; hence, a 2:4 ratio of embryo:endosperm peaks is observed. In other words, the peak ratios of 2C:3C and 2C:4C in histograms are corresponding to sexual reproduction and gametophytic apomixis without pseudogamy, respectively (see Matzk *et al.* 2000; Krahulcová and Suda 2006; Hao and Shen 2009; Hojsgaard *et al.* 2014a; Hojsgaard and Hörandl 2019 for further developmental pathways).

Asteraceae is one of the largest family of angiosperms, presenting ~24 000 species distributed across all continents excluding Antarctica (Funk *et al.* 2009). Species of Asteraceae are proportionally over-represented within the invasive plant species found in China. The percentage of invasive species of Asteraceae in China (ca. 20 %; Xu and Qiang 2018; Ma and Li 2018) is much larger than the proportion of the global flora that Asteraceae represent (8.4 %; Pyšek 1998). Moreover, the percentage of Asteraceae species invasive in China that are capable of forming an autonomous seed set (including autogamous and apomictic species) is higher than expected (65.7 %), and these self-compatible species are more widespread in China than the self-incompatible species (Hao *et al.* 2011). The family is characterized by the presence of a capitulum, surrounded by bracts, connate anthers forming a ring and fruits called cypselae (achene) (Funk *et al.* 2009). Asteraceae is commonly listed, along with Poaceae and Rosaceae, as one of the principal families within which apomixis is prolific (Czapik 1996; Noyes 2007; Hojsgaard *et al.* 2014b). Czapik (1996) reported that 44 genera in 15 Asteraceae tribes are capable of apomixis, whereas Noyes (2007) reported that apomixis occurs in 22 genera belonging to seven Asteraceae tribes (Lactuceae, Gnaphalieae, Astereae, Inuleae, Heliantheae, Madieae and Eupatorieae) in one subfamily (Asteroideae). The most recent update on apomixis in angiosperms recognized 54 genera capable of apomixis within Asteraceae (see apomixis database at www.apomixis.uni-goettingen.de) (Hojsgaard *et al.* 2014b). Of note, many apomictic Asteraceae taxa are invasive worldwide (Dellinger *et al.* 2016). In addition, this family accounts for a large proportion of invasive alien plants in China, and the majority of these invasive species are able to perform autonomous seed production by autogamy and/or apomixis (Hao *et al.* 2011). Therefore, it can be hypothesized that apomixis contributes to the invasion potential of alien Asteraceae species. Unfortunately, few studies have focused on this hypothesis to date.

The *Praxelis* genus was separated from the older and much broader *Eupatorium* genus in 1970 to form part of the Praxelinae subtribe, in which the chromosome base number is 10 (x = 10) and many species exhibit polyploidy and irregular meiosis (King and Robinson 1970). Polyploidy accompanied by apomixis is common and has been documented in a handful of Eupatorieae species (see Nogler 1984; Coleman and Coleman 1984, 1988; King and Robinson 1987; Coleman 1989; Watanabe *et al.* 1995; Bertasso-Borges and Coleman 1998, 2005; Lu *et al.* 2008; Farco *et al.* 2012).

*Praxelis clematidea* (*Eupatorium clematideum* or *E. catarium*) is an annual or short-lived perennial plant of the Eupatorieae tribe within the Asteraceae family (Veldkamp 1999; Waterhouse *et al.* 2003; Zhou 2012; Xu and Qiang 2018). The species is neotropical and originated in South America, is mainly found in southern Brazil, Venezuela, Bolivia and northern Argentina and is naturalized in Pacific and Pacific Rim areas such as Palau, East Asia and Australia (Veldkamp 1999; Ma and Li 2018; US Forest Service 2018; Xu and Qiang 2018). *Praxelis clematidea* was unintentionally introduced to China through imported ornamental plants and has since become widely distributed in southern, Southwestern and eastern areas of China such as Macao, Taiwan, Hong Kong, Fujian, Hainan, Jiangxi, Guangdong, Guangxi, Sichuan, Yunnan, Guizhou and Zhejiang (Ma and Li 2018; Xu and Qiang 2018). It is classified as one of the most serious invasive alien plants in China, and its immediate control is urgently needed (Ma and Li 2018). The species is triploid with 2n = 30 chromosomes (Watanabe *et al.* 1995). Some studies have explored apomixis in other *Praxelis* species, such as *P. pauciflora* (*Eupatorium pauciflora*) and *P. intermedia* (*E. intermedia*) (Bertasso-Borges and Coleman 1998). However, there have been no previous reports of apomixis in *P. clematidea*. Little attention has been paid to the association between seed production by apomixis and the invasive behaviour of *P. clematidea*.

In the present study, we documented seed formation in invasive alien Chinese populations of *P. clematidea* in order to answer the following questions: (i) Are these plants reproducing sexually? (ii) Is gametophytic apomixis present? (iii) Is pollination required for successful seed set? It is hoped that this study will form the theoretical foundation to understand the mechanisms behind the rapid propagation and range expansion of *P. clematidea* in order to adopt scientific and efficient control measures.

## Materials and Methods

### Reproductive pathway assessment

The reproductive pathway of *P. clematidea* was evaluated using the FCSS procedure outlined in the studies by Matzk *et al.* (2000) and Krahulcová and Suda (2006), with minor modifications. Five hundred or so mature cypselae (the indehiscent, single-seeded achenes found in Asteraceae) were collected from 30 individuals from Qingyuan (24°18’N, 113°39’E), Maoming (22°15’N, 110°55’E) and Qinzhou (21°56’N, 108°39’E) plant populations between November 2016 and January 2017. Because the individual cypselae were too small to enable single-seed analysis (the 1000-grain weights for the Qingyuan, Maoming and Qinzhou populations were 0.175, 0.200 and 0.150 g, respectively), bulked samples of 30 cypselae per population were randomly selected and used in the FCSS analysis. Cypselae were dried for several days at 40 °C in an electrothermal incubator and were placed in a blender (Joyoung JYL-D020, Jinan, China) in order to be powdered using the cutting mode for 8–15 s. The ground cypselae were then placed into a Petri dish. After adding 200 μL nuclear extraction buffer and polyvinylpyrrolidone (PVP), 800 μL 4’,6-diamidino-2-phenylindole (DAPI) staining buffer was added, and the Petri dish was covered with an aluminium foil. After filtering using 30-μm filters (Celltrics, Partec, Germany), Ploidy Analyser II (Partec, Germany) and the FCSS Express 6 Plus software package (De Novo Software) were used to analyse the reproduction pathway according to the relative positions of the embryo and endosperm peaks of the mature cypselae presented on the histograms of the Partec flow cytometer and according to the methodology of Matzk *et al.* (2000).

To exclude the possibility of ploidy shifts in the embryo due to partial apomixis (e.g. Schinkel *et al.* 2017), the ploidy levels of fresh leaves and dry cypselae from the same individuals were analysed via Partec flow cytometry, using leaf ploidy as an external standard for embryo ploidy. Five families, each represented by a 1-cm^2^ sample of fresh leaves and a bulk sample of 30 mature dry cypselae from the same individual from Guangzhou, Guangdong, China (23°10N, 113°20E) were selected randomly and used for flow cytometry in July 2020. CyStain® UV Precise P kits were used to prepare samples of leaves and cypselae. For each family, the measurement parameters of the peak positions of the mother plant leaf samples were immediately used to measure the cypselae samples. The Ploidy Analyzer II and the CyView™ V.1.6 software package were used to determine the ploidy levels of leaves, embryos and endosperm. Peak indices 1 (PI1; mean peak value of leaves compared to the mean peak embryo value) and peak indices 2 (PI2) (mean peak embryo value compared to the mean endosperm peak) were assessed.

### Megagametophyte and embryo development

In total, 5–10 inflorescences or infructescences at different developmental stages were collected from 15 to 30 *P. clematidea* plants and were fixed to observe megagametophyte development in October 2018 in Zhuhai (22°15’N, 113°31’E) and in April 2019 in Guangzhou (23°10’N, 113°20’E). The collected *P. clematidea* plants were growing at least 10 m away from one other. Observations of megagametophyte development in ovules were conducted between March and April 2019. There were 45–60 florets in each inflorescence, and in total, 80 inflorescences or infructescences and 10 florets per inflorescence or infructescence were observed. The fresh capitula collected at different flower bud, flower and cypselae developmental stages were immediately fixed in a mixed solution of formalin-acetic-alcohol 70 % (FAA 70) (Johansen 1940). After being taken to the laboratory, the fixed capitula were dehydrated with different concentrations of ethanol before being cleared through methyl salicylate (Sigma-Aldrich, St Louis, MO, USA) using the whole-mount clearing method (Herr 1971, 1974; Young *et al.* 1979; Hao and Qiang 2007; Hojsgaard *et al.* 2014a). The cleared capitula were placed onto Raj slides. Ten florets per capitulum were separated on the slides using a dissecting needle and tweezers under an anatomical microscope. A drop of methyl salicylate was added to prevent the florets from drying before the slides were covered. Ovule and embryo development as well as pollen morphology were observed and recorded using an Olympus BX53 microscope with DIC optics (Olympus, Tokyo, Japan) and a camera of VIS 500C (Weihan, Shanghai, China) at ×100–×1000 magnification. The main stages of megagametophyte and embryo development were photographed. To determine the presence or absence of a callose wall in the MMCs, 20 florets from five young capitula were taken from the FAA 70 fixation solution and were prepared according to the following steps: florets were rinsed with 70, 50 and 30 % ethanol in addition to distilled water, they were then cleared in 8 N NaOH at 60 °C for 20 min before being rinsed several times with tap water, and were then rinsed again with Tris-glycine buffer (pH 8.3). Cleared florets were stained with water-soluble aniline blue (0.1 % aniline blue in 0.1 M K_3_PO_4_) (Martin 1959). The MMCs were examined using a light field microscope (Nikon ECLIPSE90i), and callose deposition in the cell walls was examined using fluorescence in the same location. The figures were organised using Adobe Photoshop CS6 (Adobe, California, USA).

### Pollen viability and size

Ten *P. clematidea* individuals were transplanted to the lab of Guangzhou Customs Technology Center so that fresh florets could be easily got in July and August 2020. The 10–15 disk florets from each individual were taken randomly from different branches and capitula before flowering. Pollen viability was quantified using the following two methods.

#### Benzidine test.

Pollen viability was assessed through the oxidation of benzidine by peroxidase in the presence of hydrogen peroxide (Kong and Yi 2008). Two solutions were freshly prepared: (i) 0.5 % benzidine dissolved in 50 % ethanol, 0.5 % α-naphthol dissolved in 50 % ethanol and 0.25 % sodium carbonate were mixed with equal volumes before use (Solution 1), (ii) 0.3 % hydrogen peroxide (Solution 2). The anthers of the disk florets were cut and placed into a mixed solution of Solution 1 and Solution 2 (10 µL each) on the slide and were then mashed with a glass rod. Many oxygen bubbles were released and observed due to catalase activity. The slides were covered by a coverslip and placed in Petri dishes on two 2-layers of wet filter paper. Then, the Petri dishes were covered and placed in a growth chamber at 35 °C. After 30 min, the pollen was viewed under a Zeiss Axio Image Z1 microscope (Carl Zeiss AG, Oberkochen, Germany) at ×200 or ×400 magnification. Dead pollen remains colourless or yellow, whereas live pollen turns purplish red. All visible pollen grains were measured and photographed using AxioVision 15.0 software (Carl Zeiss AG, Oberkochen, Germany). In total, 4493 pollen grains from seven individuals (167–1741 grains per individual) were counted and statistically analysed. Pollen size was measured and analysed for a total of 187 pollen grains from eight individuals, for 3–83 pollen grains per individual.

#### Alexander’s stain.

Pollen fertility was assessed by staining the pollen walls and cytoplasm, according to the methodology of Alexander (1969). The staining solution contained three dyes: Malachite green (Merck, MKCJ3206, America), Acid fuchsin (Sinopharm Chemical Reagent Co., Ltd, 71019360, Shanghai, China) and Orange G (Merck, SHBL4754, America). The anthers of the disk florets were cut and put into the staining solution on the slide. After the anthers were mashed using a glass rod and covered by a coverslip, the slides were placed in Petri dishes on two 2-layers of wet filter paper. Then, the Petri dishes were covered and placed in the growth chamber at 50 °C and were then viewed under the Zeiss Axio Imager Z1 microscope at ×200 or ×400 magnification. The pollen grains that were green in colour were aborted, whereas pollen grains that were red in colour were non-aborted. The available pollen grains were measured and photographed using AxioVision 15.0 software (Carl Zeiss AG, Oberkochen, Germany). In total, 1052 grains from four individuals, for 41–711 pollen grains per individual, were counted and analysed.

### Pollen morphology

Ten medium-sized capitula (before or after the first day of flowering) were taken from the FAA 70 solution. The samples were then dehydrated using a graded ethanol series and were cleared with xylene. Subsequently, the florets were embedded in paraffin and spliced into 8–10 μm longitudinal sections on a rotary microtome (Leica RM2125). The anthers and pollen were examined using a Nikon ECLIPSE90i microscope under bright and fluorescent light sources after staining with water-soluble aniline blue or DAPI-containing Vectashield mounting medium (Vector Laboratories, H-1200), respectively.

### Breeding-system assessment

From November 2016 to March 2018, mature *P. clematidea* cypselae were collected from natural populations in Zhuhai (22°16N, 113°33E), Maoming (22°15N, 110°55E), Jiangmen (22°10N, 112°22E) and Qinzhou (21°56N, 108°39E) in order to evaluate pollination. The cypselae collected from the four populations were sowed in seedling-raising equipment within a tissue culture room after gibberellin treatment. The germinated seedlings (4–7 individuals per population) were transplanted into pots after 20 days and were placed in the garden of Changshu Institute of Technology (31°36′N, 120°43′E). After flowering, three capitula from the same individual were treated as one group for the following three experimental treatments: pollination, bagging and emasculated capitula, following the procedures customized for emasculating Asteraceae individuals (Richards 1997; Hao *et al.* 2011). For the emasculation treatment, the anthers, upper part of the style and stigma of all florets within the capitula were cut off with sharp scissors without harming the ovary or ovules. Fifteen groups of mature capitula with achenes were harvested per population from different individuals, and the seed set was calculated as the proportion of florets per capitulum that developed achenes, using the following formula. The breeding system was assessed by comparing the seed set among different treatments.

$$\begin{array}{l} Seed\;set=\frac{Number\;of\;mature\;seeds\;per\;capitulum}{Total\;number\;of\;florets\;per\;capitulum} \end{array}$$

### Statistical analyses

After arcsin transforming the seed set percentages, significant differences in the seed set among different treatments and populations were tested using SPSS data analysis software 16.0 (IBM SPSS Statistics). Differences found using Tukey or Dunnett’s C tests of one-way and two-way analysis of variance (ANOVA) analyses were classified as significant when the *P*-values of the test of homogeneity of variances were ≤0.05 or >0.05, respectively. The geometric mean amount of DNA between leaves and embryos was tested using the *t*-test method of pair-samples to show whether the ploidy of the embryo shifted from the mother plant to siblings. One-sample *t*-tests were used to test the peak indices of PI1 (test value = 1) for five individuals from the Guangzhou population and of PI2 (test value = 2) for eight bulked seed samples (five samples from the Guangzhou population and three samples, i.e. one each, from the populations from Qingyuan, Maoming and Qinzhou). The mean and test values of PI1 and PI2 were regarded as insignificantly different when the *P*-value of test of variances was >0.05. The Origin Pro 9.0 software package (OriginLab Corporation, Northampton, MA, USA) was used to generate histograms of seed sets for different treatments within the same populations and for different populations subjected to the same treatment. Significant differences are marked with different letters.

## Results

### Reproductive pathway

**Figure 1.**
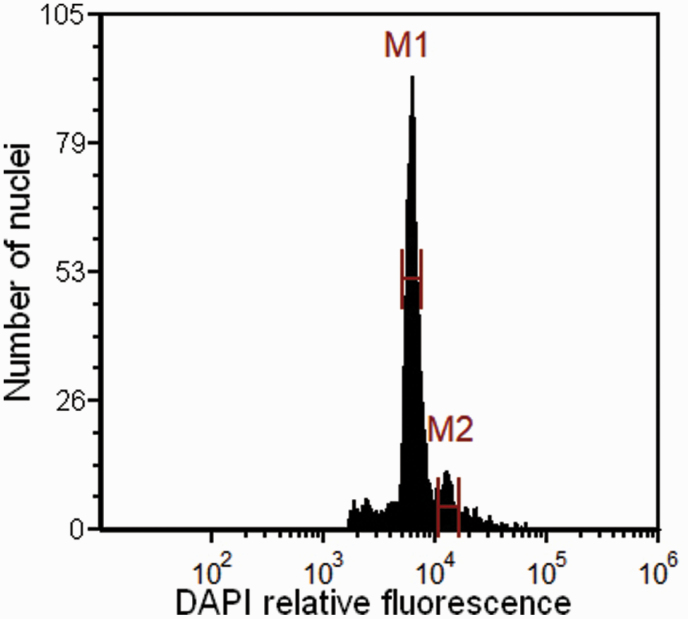
Flow cytometric seed screening of *P. clematidea* showing peak values of embryo (2C) and endosperm (4C).

In total, 90 mature cypselae were analysed. After adjusting the embryo peak of *P. clematidea* to the position of 2C, the endosperm peak was located at position 4C (Fig. 1). The ratios of the relative positions of the embryo and endosperm peaks were 2C:4C (Fig. 1). The PI1 was 1.01 ± 0.03 (Table 1; **see****Supporting Information 1—Tables S1**–**S3**, **Supporting Information 2**), and the paired-samples *t*-tests for geometric mean DNA contents (Table 1) showed no significant differences (*t* = 0.939, df = 4, *P* = 0.401) between leaves and embryos across the five tested families. The result of one sample t-test also revealed no ploidy shifts in *P. clematidea* embryos (Table 1). The PI2 was 2.02 ± 0.04 (Table 1; **see****Supporting Information 1—Tables S2****and****S3**, **Supporting Information 2**) and one-sample t-test showed that there were no significant differences in the results obtained against the expectation of 2:1 from eight bulked seed samples from four populations (Table 1). The results suggested that the reproductive mode of *P. clematidea* was autonomous gametophytic apomixis without pseudogamy. Pollen and fertilization were not needed for *P. clematidea* seed formation, that is, both the embryo and endosperm were parthenogenetic products of unreduced eggs and central cells.

**Table 1.** One-sample *t*-tests of PI1 and PI2, showing the same ploidy level of mother plant (leaves) and embryos (PI1) and the expected 1:2 ratio of embryo:endosperm (PI2). *Peak indices 1 (PI1) mean peak value of leaf compared to the mean peak of embryo. The peak indices 2 (PI2) indicate the peak value of the embryo compared to the mean peak of the endosperm.

Peak indices (PI)*	Test value	Mean	SD	*t*	df	*P*	Mean difference
PI1	1	1.01	0.03	0.967	4	0.388	0.012
PI2	2	2.01	0.04	0.970	7	0.365	0.015

### Formation of the megagametophyte of *Antennaria*-type diplospory

**Figure 2.**
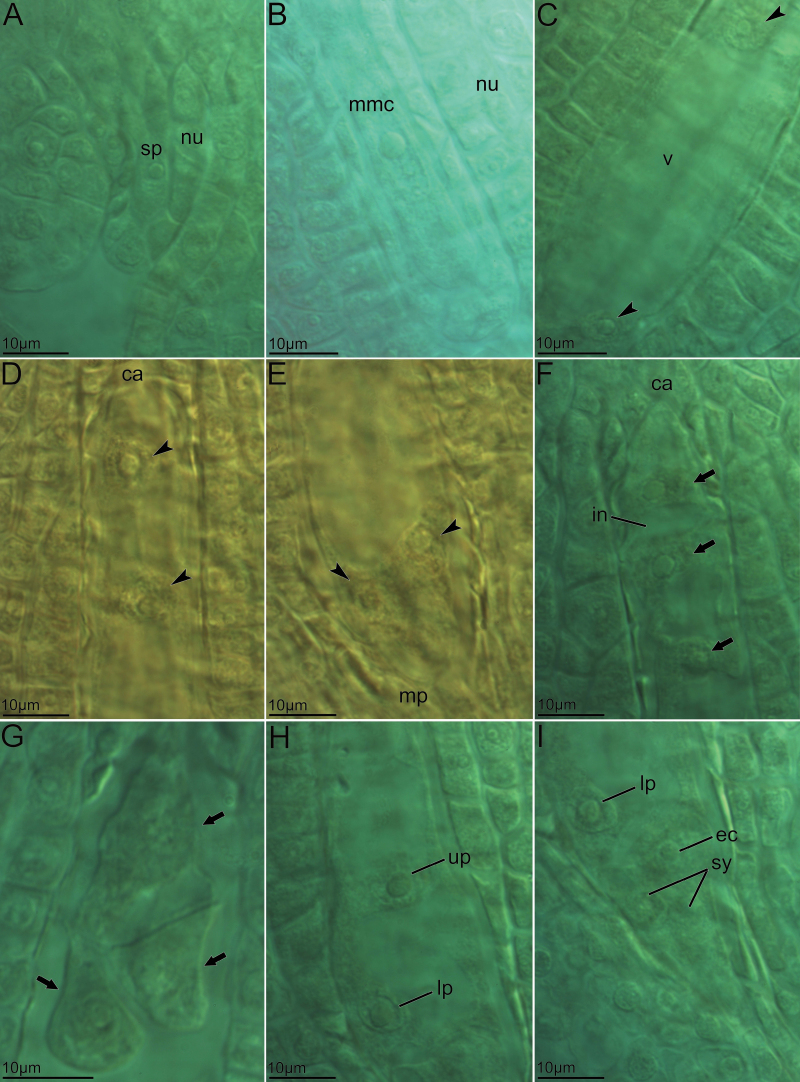
Embryological development of the *Antennaria*-type embryo sac of *P. clematidea*. (A) Sporogeneous cell. (B) Megaspore mother cell stage. (C) Two-nucleate megagametophyte. (D, E) Four nucleate megagametophyte filled with large vacuoles, presenting two nuclei at the chalazal pole (D), and two nuclei at the micropylar pole (E). (F–I) Mature megagametophyte with seven cells and eight nuclei. (F) Antipodal cell containing two cells and three nuclei at the chalazal pole. (G) Antipodal cell containing three cells. (H) Two polar nuclei. (I) Egg apparatus and lower polar nucleus. Egg apparatus was comprised of one egg cell and two synergids. Arrow, antipodals; arrowhead, nuclei of immature megagametophyte; ca, chalazal end; ec, egg cell; in, intercellular space; lp, lower polar nucleus; mmc, megaspore mother cell; mp, micropylar end; nu, nucellus; sp, sporogenous cell; sy, synergids; up, upper polar nucleus; v, vacuole.

The capitula of *P. clematidea* are homogamous and contain 35–40 disk florets. The florets of *P. clematidea* possess an inferior and unilocular ovary, in which a single ovule is located on the basal placenta. The ovule of *P. clematidea* is anatropous and tenuinucellate. At the early stage of development, when the anatropous ovule was not fully formed, an archesporial cell (Fig. 2A) was observed to differentiate under the nucellar epidermis at the micropylar pole. After a short transition period, the archesporial cell expanded into a narrow and long MMC (Fig. 2B). No callose was detected in the MMC wall under refluorescence microscopy after the cells were stained with water-soluble aniline blue under UV excitation light. Two small vacuoles formed at each pole of the MMC. The MMC underwent a long interval without meiosis, containing one nucleus and two large vacuoles after intense vacuolization of the cytoplasm. Because MMCs omit meiosis, their function as initial cells for megagametophyte development is similar to that of an unreduced megaspore, which results from restitutional meiosis. No tetrads were observed in any of the florets. After vacuole formation, the MMC further enlarged along the axis of the micropyle–chalaza, and the two vacuoles at both poles became larger. With the division of the nucleus, a two-nucleate cell was formed (Fig. 2C). The two nuclei then moved from the centre of the megagametophyte to the different poles. The two vacuoles fused into one larger vacuole, which filled the megagametophyte, and this was clearly visible. Then, each nucleus located at the micropyle and chalazal poles of the developing megagametophyte underwent a second mitosis, resulting in a four-nucleate megagametophyte that was larger than the two-nucleate megagametophyte and was filled with a large vacuole (Fig. 2D and E).

After the four-nucleate stage, each nucleus underwent a third mitosis and formed an eight-nucleate megagametophyte. The volume of the megagametophyte expanded rapidly along the longitudinal axis. Shortly after the formation of the eight-nucleate megagametophyte, one nucleus each from the chalazal and micropylar poles moved separately towards the centre of the megagametophyte and formed two polar nuclei. Cell walls formed around each nucleus (except the two polar nuclei) and a seven-celled megagametophyte thus appeared (Fig. 2F–I). There were two kinds of antipodal cell types: one was a three-cell type without vacuoles in the antipodal cells (Fig. 2F), and the other was a two-cell type with intense vacuolation in each antipodal cell. One antipodal cell far from the chalazal pole contained two nuclei, and the other one contained one nucleus (Fig. 2G). The majority of the cytoplasm, including the vacuoles of the megagametophyte, was enclosed by the central cell with the largest volume in the embryo sac (Fig. 2H). Three cells at the micropylar pole developed into an egg apparatus with one egg cell and two synergid cells (Fig. 2I). Therefore, the eight-nucleate megagametophyte developed without meiosis and formed according to *Antennaria*-type diplosporous apomixis.

### Parthenogenesis of embryos and endosperm

**Figure 3.**
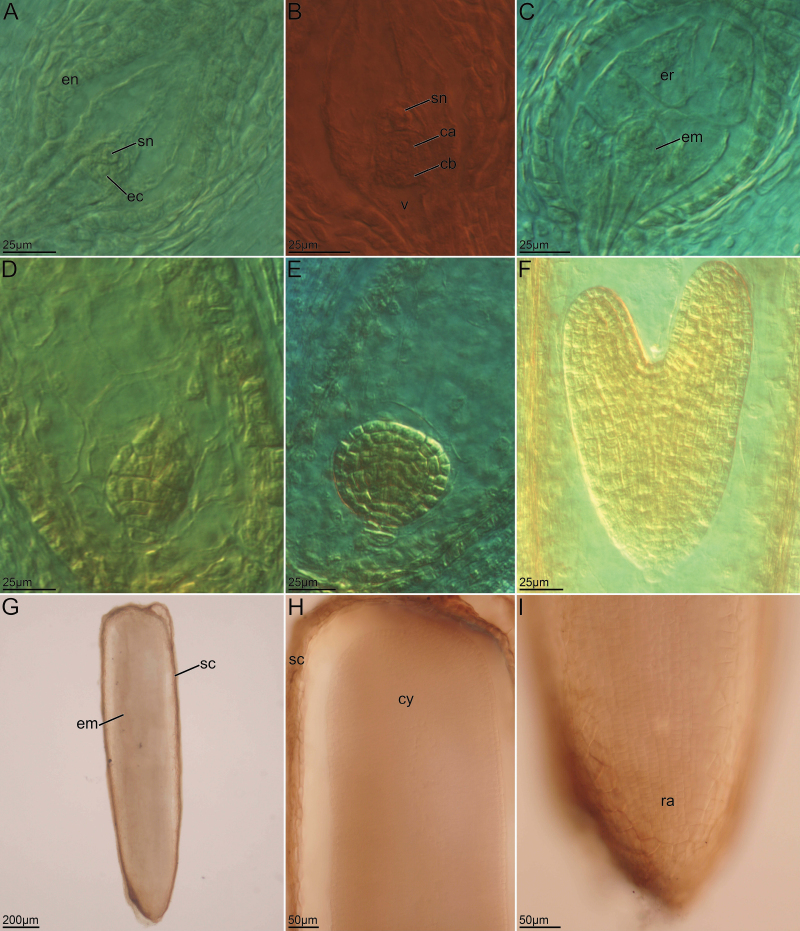
Development of embryo and endosperm of *P. clematidea*. (A) Two polar nuclei fused into a secondary nucleus near the egg cell. (B) The egg cell divides into a two-celled proembryo before the secondary nucleus develops without fertilization (containing an apical cell and a basal cell). (C) Multicellular embryo and cellular-type endosperm. (D, E) Globular embryo. (F) Heart-shaped embryo. (G) Mature embryo inside seed coat. (H, I) Details of the G image, indicating the region of the cotyledon (H) and radicle (I), respectively. ca, apical cell; cb, basal cell; cy, cotyledons; ec, egg cell; em, embryo; en, endothelium; er, endosperm; ra, radicle; sn, secondary nucleus; sc, seed coat; v, vacuolation.

In the mature eight-nucleate megagametophyte, the two polar nuclei gradually approached one another and fused into a secondary nucleus. At this stage, the synergid cells tended to degenerate. Nucellar cells around the megagametophyte were absorbed, resulting in an enlarged megagametophyte (Fig. 3A). Then, the unreduced egg cell began to divide into a two-celled proembryo without fertilization, which occurred before the division of the primary endosperm nucleus; there was a distinct vacuole near the micropylar pole (Fig. 3B). The two-celled proembryo underwent further mitotic divisions, and endosperm development was cellular (Fig. 3C and D). The proembryo continued to divide to form a globular embryo with a distinct suspensor connected to the endothelium (Fig. 3E) before developing into a heart-shaped embryo (Fig. 3F) and finally into a mature embryo (Fig. 3G–I). The endosperm continued to divide, presenting highly vacuolated, large and loose cells. During embryo development, both apical and basal cells were involved in embryo formation, conforming to *Asterad*-type embryo development. In this process, both the embryo and endosperm developed without fertilization, embryos developed parthenogenetically from the egg cell and the endosperm developed autonomously from polar nuclei without fertilization, that is, *P. clematidea* is diplosporous with autonomous apomixis.

### Pollen viability and size

**Figure 4.**
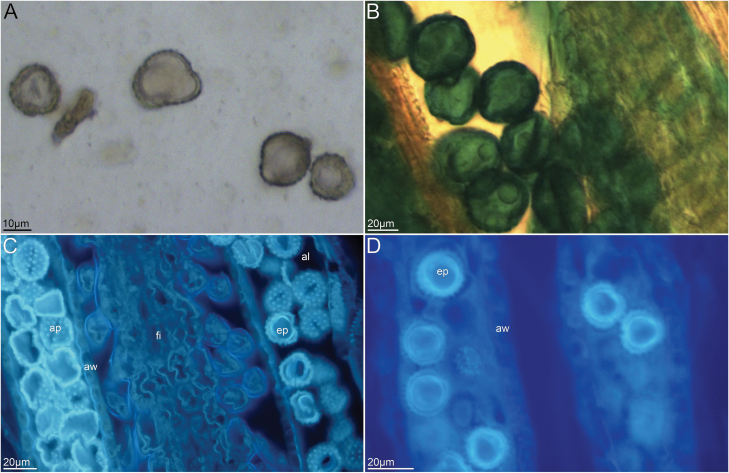
Various forms of pollen grains of *P. clematidea*. (A) Non-viable pollen grains stained with benzidine. (B) Aborted pollen grains stained with Alexander’s method. (C) Aborted pollen grain stained with water-soluble aniline blue under fluorescent microscope. No callose fluorescence was observed inside of the pollen grains. Usually, callose is present in the wall of primary generative cells of fertile pollen grains. Autofluorescence of the sporopollenin on the outer wall and excited fluorescence from callose on the inner wall of disintegrated pollen grains were observed. (D) Aborted pollen grain stained with DAPI under fluorescent microscope. Neither nuclei from vegetative cell nor from generative cell could be observed. Weak fluorescence was from the outer wall and the inner wall of pollen grains. ap, aborted pollen grain; aw, anther wall; al, anther locule; ep, empty pollen grain; fi, filament.

Pollen viability was 0.82 ± 0.57 % (mean ± SD) **[see****Supporting Information 3****]** and 0.36 ± 0.44 % (mean ± SD) **[see****Supporting Information 4****]** as measured by the Benzidine test (Fig. 4A) and Alexander’s stain (Fig. 4B), respectively. Pollen grains measured 17.93 ± 2.70 µm in size **[see****Supporting Information 5****]**.

### Pollen grain morphology

The present study found that the majority of pollen grains in *P. clematidea* were aborted. Pollen grains were empty and neither cytoplasm nor nuclei were found within the pollen grains, as observed after staining with water-soluble aniline blue and DAPI (Fig. 4C and D). This further proved the occurrence of autonomous apomixis in which the embryo and the endosperm can develop independently of fertilization in *P. clematidea.* Bad pollen quality also corroborated that *P. clematidea* exhibited a reproductive pathway of autonomous apomixis without pseudogamy as observed via FCSS.

### Breeding system

**Figure 5.**
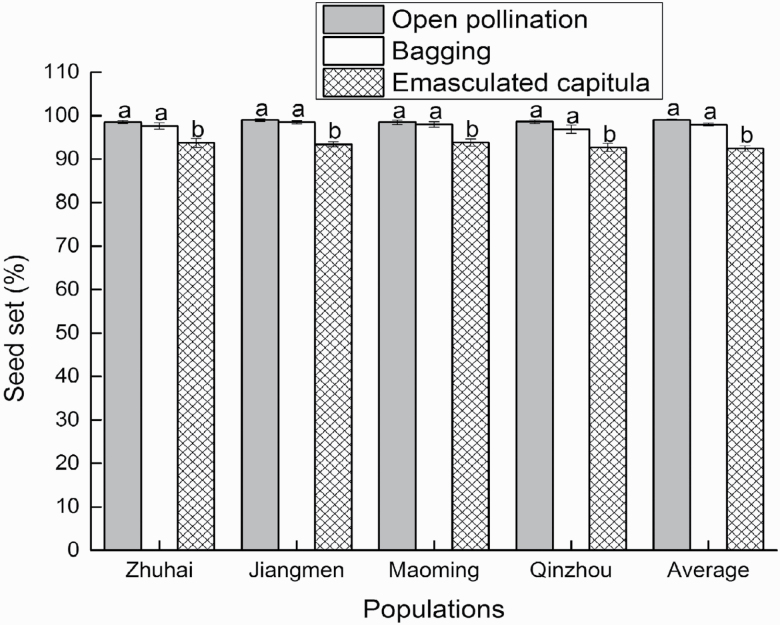
Seed set among three treatments for four populations. Each value is the mean percentage (SD) of 15 replications, and statistical analysis unit is each capitulum. Different lowercase letters represent significant differences among treatments in same population (*P* < 0.05). Same lowercase letters represent not significant differences among treatments in same population (*P* > 0.05).

The seed set values of the open-pollination, bagging and emasculated capitula treatments were all >90 % (Fig. 5; **see****Supporting Information 6—Sheets 1****and****2**). One-way ANOVA of the seed set of different treatments within the same population showed that there was a significant difference among the three treatments (Table 2). Although the seed set of the emasculation treatment (93.4 ± 0.4 %) was significantly less than the seed sets of open pollination (98.7 ± 0.2 %) and bagging (97.7 ± 0.4 %), the seed set was still >90 % (Fig. 5). The experiment showed that pollen and fertilization were not needed for seed formation in *P. clematidea*, that is, both the embryo and endosperm were parthenogenetic products of unreduced eggs and central cells. This demonstrated that the breeding system of *P. clematidea* was autonomous apomixis, which was corroborated by the FCSS and megagametophyte formation results.

**Table 2.** Analysis of variance of pollination experiments.

	Test of homogeneity				ANOVA				
	Levene statistic	df1	df2	*P*	Sum of squares	df	Mean square	*F*	*P*
Among treatments									
Zhuhai	4.304	2	42	0.020	0.371	2	0.186	10.964	<0.0001
Maoming	4.226	2	42	0.021	0.441	2	0.221	13.913	<0.0001
Jiangmen	3.716	2	42	0.033	0.570	2	0.285	33.419	<0.0001
Qinzhou	2.526	2	42	0.092	0.505	2	0.252	12.783	<0.0001
Average	11.599	2	177	<0.0001	1.866	2	0.933	63.486	<0.0001
Among populations									
Open pollination	1.655	3	56	0.187	0.007	3	0.002	0.188	0.904
Bagging	2.297	3	56	0.088	0.019	3	0.006	0.282	0.838
Emasculated capitula	2.228	3	56	0.095	0.010	3	0.003	0.329	0.804
Among treatment * populations									
	6.105	11	168	0.000	14.700	6	2.450	0.350	0.909

**Figure 6.**
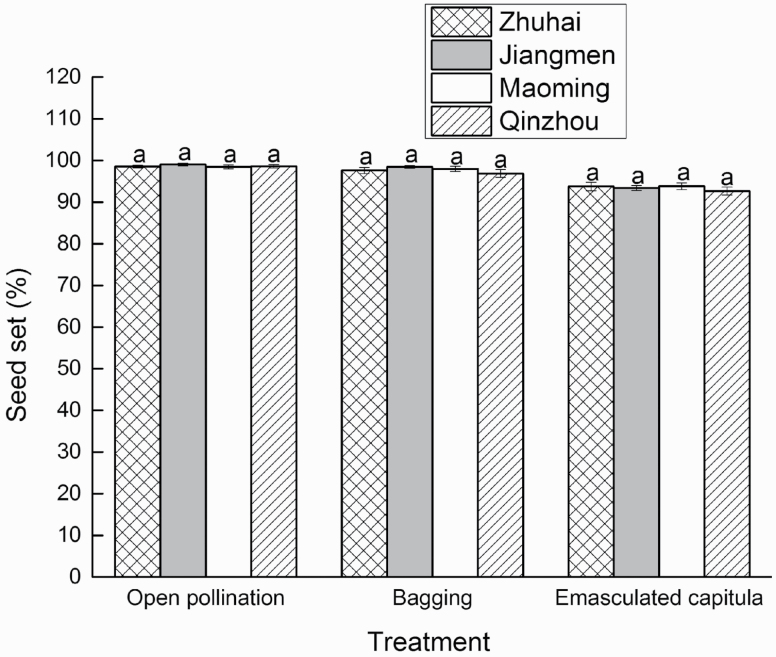
Seed set among four populations for three treatments. Different lowercase letters represent significant differences among populations in same treatment (*P* < 0.05). Same lowercase letters represent not significant differences among treatments in same population (*P* > 0.05).

Comparisons among populations subjected to the same treatment revealed that there were no significant differences among the four populations for any of the three treatments (Table 2; Fig. 6; **see****Supporting Information 6—Sheets 1****and****3**). There was also no significant effect of the treatment * population (*F* = 0.350, df = 6, *P* = 0.909) interaction, as determined by two-way ANOVA (Table 2). This indicates that autonomous apomixis is a stable trait for *P. clematidea* in China.

## Discussion

### Apomixis in *P. clematidea*

The results of the FCSS, embryological development, pollen viability, and pollination experiments in the present study clearly demonstrate that *P. clematidea* exhibits diplosporous apomixis without fertilization of egg cells in China. The absence of meiosis during megasporogenesis, *Antennaria*-type embryo sac development, parthenogenetic embryo development, autonomous endosperm development and sterile pollen were observed.

Bertasso-Borges and Coleman (1998) have also reported that the mode of embryo sac development utilised by the congeneric species *Eupatorium pauciflorum* (= *Praxelis pauciflora*) is diplosporous apomixis. Similar to the observations in the present study, the authors of the aforementioned study observed precocious formation of the embryo and endosperm, the absence of pollination and the triploidy of *P. pauciflora*. Triploidy, polyploidy or aneuploidy is often associated with meiotic failure resulting from unbalanced chromosome pairing and segregation, which may trigger apomixis. Examples of the association between diplosporous apomixis and triploidy in Asteraceae species include *Erigeron annuus* (McDonald 1927; Noyes 2007) and other weedy segregates of broader the *Eupatorium* genus, such as *Ageratina adenophora* (= *Eupatorium glandulosum*, see Nogler 1984 and *E. adenophora*, Lu *et al.* 2008), *E. bupleurifolium*, *E. callilepis* (*= Chromolaena callilepis*) (Coleman and Coleman 1984), *E. squalidum* (*= C. squalidum*) (Coleman and Coleman 1988) and *P. pauciflora* (= *E. pauciflora*, Bertasso-Borges and Coleman 1998). The association between diplosporous apomixis and autohexaploidy has also been reported in *E. odoratum* (*= Chromolaena odoratum*) (Coleman 1989) and *E. laevigatum* (Bertasso-Borges and Coleman 2005). Moreover, the association between apospory and triploidy has been reported in another perennial Eupatorieae herb, *Campuloclinium macrocephalum* (Farco *et al.* 2012). This association with polyploidy has been thought to be caused by the expression of recessive lethal genes linked to apomixis genes in the megagametophyte (Nogler 1984; Richards 1996). More recent studies have explained apomixis as the result of the differential, ectopic or asynchronous expression of genes that normally regulate the sexual pathway (Koltunow and Grossniklaus 2003; Hand and Koltunow 2014). Apomixis likely starts in diploid plants and is enhanced by polyploidy (Hojsgaard and Hörandl 2019).

According to previous studies, gametophytic apomixis is confined to perennial plants and occurs very rarely in annual apomicts (Bierzychudek 1985; Asker and Jerling 1992). *Praxelis clematidea* was an annual weed in the present study. However, different life forms have been reported for this species in different countries, where it ranges from an annual, short-lived perennial (Waterhouse *et al.* 2003; Zhou 2012; Xu and Qiang 2018) to a suffrutescent shrub (Abbott *et al.* 2008; US Forest Service 2018). Moreover, unlike other triploid apomicts, which are mainly restricted to plants with *Taraxacum-* and *Ixeris*-type diplospory (Asker and Jerling 1992), *P. clematidea* displays *Antennaria-*type diplospory, in which unreduced embryo sacs and seeds are formed only by mitosis, without any restitutional nuclei. This is the first report of *Antennaria-*type diplospory in a triploid apomict. These specific traits of diplosporous gametophytic apomixis in annual *P. clematidea* are likely associated with its rapid production of seeds, which allows the species to quickly colonise new areas. Large numbers of seeds can be produced in as few as three or four months after germination (Waterhouse *et al.* 2003). Further studies are necessary to investigate whether the reproductive mode and type of apomixis differ among populations with different life forms and histories.

The FCSS results revealed two peaks with a 2C:4C ratio. This result can be interpreted as either confirming apomixis with a 2C embryo and autonomous endosperm formation (i.e. without fertilization of the two unreduced polar nuclei by sperm nuclei) or as a G2 peak of the growing embryo. In the latter case, the endosperm may be missing or small and already consumed, as is typical in Asteraceae (Noyes 2007). The present authors believe the first interpretation to be more likely because all cypselae were fully mature and always displayed the second peak at the 4C position. G2 peaks usually appear only occasionally in immature seed material when some cells of the embryo are still in the synthesis phase before mitosis. Autonomous apomixis is the rule in Asteraceae, and only a few genera exhibit pseudogamy (Noyes 2007). Furthermore, the observed aborted pollen grains indicate that fertilization of the egg and polar nuclei is no longer possible in *Praxelis*. Consistent with pollen-independent apomixis, no ploidy shifts in the embryo were observed, which otherwise often occur due to fertilization of unreduced egg cells by one or more sperm nuclei (so-called BIII hybrids) (Nogler 1984; Bicknell and Koltunow 2004; Schinkel *et al.* 2017). With pollen viability below 1 %, any male contribution to offspring is negligible. Similarly, dramatic reductions in pollen fertility, or no pollen at all, have been reported in other contribal apomixis weeds of Eupatorieae such as *E. bupleurifolium*, *E. callilepis* (*C. callilepis*), *E. squalidum* (*C. squalidum*), *E. odoratum* (Coleman and Coleman 1984, 1988; Coleman 1989), *E. pauciflorum* (*Praxelis pauciflora*), *E. intermedium* (*P. intermedium*) (Bertasso-Borges and Coleman 1998), *E. laevigatum* (Bertasso-Borges and Coleman, 2005) and *Campuloclinium bupleurifolia* (Farco *et al.* 2012). The total absence of germinated pollen grains on exposed stigmas was also observed in another invasive *Eupatorium* weed (*A. adenophora*) in China (Lu *et al.* 2008). The irregular pairing of chromosomes during meiosis is the reason for pollen sterility (Watanabe *et al.* 1995).

In the current study, the seed set achieved under the emasculation treatment was significantly lower than the seed sets of the open-pollination and bagging treatments. This may be due to the cutting of the upper part of the capitula, which can harm the tissue and may disturb the development of the ovaries located below the cutting site. Nevertheless, the seed set still reached >90 % in the emasculation treatment, indicating that *P. clematidea* is capable of reproduction via autonomous apomixis. A high seed set after emasculation (41 ± 17.0 %) was also reported in the apomictic *A. adenophora*, which is another serious invasive alien weed in China (Lu *et al.* 2008). The combination of pollen infertility and high seed sets in these autonomous apomixis weeds may increase the opportunities for reproducing and spreading for these invasive species.

### The association of apomixis with the invasion of *P. clematidea*

When invasive alien plants are introduced to a new habitat, reproductive assurance is a fundamental challenge for the establishment of populations (Dellinger *et al.* 2016). Introduction usually begins with a small number of individuals, and the pollination services of the native area are often not available (Prentis *et al.* 2008). Selfing is a possible solution for reproductive assurance during colonization; however, it is associated with loss of heterozygosity and inbreeding depression (Prentis *et al.* 2008). Apomixis provides reproductive assurance without the concomitant loss of heterozygosity because maternal genotypes are inherited. Pseudogamous apomixis has the disadvantage that it must be connected to self-compatibility to allow for uniparental reproduction (Hörandl 2010). Reports in the literature have stated that plants capable of autonomous apomixis have a reproductive advantage because they can produce offspring without the need to fertilize the endosperm with pollen. This enhances their capability to colonize and establish themselves in introduced areas (Hojsgaard *et al.* 2014b). *Praxelis clematidea* is a serious invasive alien species in China; its rapid dispersal is likely to be closely related to its diplosporous autonomously apomictic reproductive mode. This reproductive mode enables the species to produce large numbers of seeds and to establish and expand new populations rapidly. In addition to autonomous apomixis in *P. clematidea*, the pollination experiment carried out in the present study further demonstrated that *P. clematidea* produced a high seed set under both open-pollination and bagging treatments as well as under the emasculated capitula treatment, meaning that the plants ensured reproductive success via diplosporous autonomous apomixis. Despite being an obligate apomict, *P. clematidea* is probably not affected by a loss of genetic diversity. One recent study showed that the genetic diversity of *Eupatorium catarium* (= *P. clematidea*) populations found on Hainan island and in Guangdong in China was high according to inter-simple sequence repeat (ISSR) analysis; at the species level, the Shannon’s information index (*I*) was found to be 0.444 and Nei’s gene diversity (*h*) was measured at 0.2916 (Li *et al.* 2014). Population genetics studies have revealed that uniclonality in apomicts is rare, and that heterozygosity is usually higher than that in their sexual relatives because of hybridity and polyploidy (Hörandl and Paun 2007). Genetic diversity in apomictic populations can arise from hybrid origins, mutations, backcrossing with sexual relatives and residual sexuality (Hörandl and Paun 2007). In *P. clematidea*, the first two factors most likely contributed to genetic diversity despite obligate apomixis. Furthermore, multiple independent introductions can increase genetic diversity in invasive plant populations (Prentis *et al.* 2008), which was likely also the case for *P. clematidea*. Therefore, the probability of successful establishment and dispersal of *P. clematidea* is greatly increased, and eventually the species is able to create more and larger invasive populations in its introduced and naturalized habitats.

It was also observed that male gametophytes of *P. clematidea* were almost completely sterile, which is consistent with the results of the study by Bertasso-Borges and Coleman (1998) regarding male sterility due to microsporocyte degeneration prior to meiosis. This implies that the large-scale invasion and strong reproductive capacity of *P. clematidea* were closely related to the species’ ability to reproduce via diplosporous autonomous apomixis. In reproductive processes without male gametophytes, most resources are allocated to female gametophytes to produce offspring, and the probability of reproductive success further increases. Moreover, reproduction becomes independent from pollinators, which is also an advantage in newly invaded areas where the pollinator spectra may differ from the home range. Invasion is a complex process involving many factors, but both apomixis and self-compatibility are clearly advantageous for invasiveness (Hao *et al.* 2011; te Beest *et al.* 2012; Razanajatovo *et al.* 2016). The *Antennaria*-type diplosporous apomixis displayed by *P. clematidea* likely contributed directly and closely to its large-scale invasion and spread because apomixis can lead to the formation of successful clones, which can extend over large distribution areas (Hörandl 2008). Effective control efforts should be taken to remove above-ground plant parts and to prevent the formation of seeds, as the plant mainly spreads by cypselae and can produce plenty of cypselae quickly after germinating (Waterhouse *et al.* 2003).

## Supporting Information

The following additional information is available in the online version of this article—

**Supporting Information 1.** Raw data and Histograms of FCSS for 4 populations.

**Supporting Information 2.** Raw data of Table 1.

**Supporting Information 3.** Pollen viability of Benzidine test.

**Supporting Information 4.** Pollen viability of Alexander’s stain.

**Supporting Information 5.** Pollen size data.

**Supporting Information 6.** Raw and arcsin transformation date of Table 2.

plab007_suppl_Supplementary_Information_1

plab007_suppl_Supplementary_Information_2

plab007_suppl_Supplementary_Information_3

plab007_suppl_Supplementary_Information_4

plab007_suppl_Supplementary_Information_5

plab007_suppl_Supplementary_Information_6

## Data Availability

All data used during the study appear in the submitted article.

## Notes

**Populations & Communities. Chief Editor:** Jean Burns
