# Supplementary material for: Autonomous apomixis in Praxelis clematidea (Asteraceae: Eupatorieae), an invasive alien plant
Source: AoB Plants. 2021 Jan 29;13(2):plab007. doi: 10.1093/aobpla/plab007 (PMC8035972; doi:10.1093/aobpla/plab007)
Supplement: plab007_suppl_Supplementary_Information_1 [file plab007_suppl_supplementary_information_1.pdf]

Table 1 The FCS indices of leaf sample for 5 families of Guangzhou population.

Sample	Events	Geometric	CV
Leaf 1	1242	6394.47	4.15
Leaf 2	1316	6672.66	4.76
Leaf 3	1552	6623.49	5.79
Leaf 4	1531	6463.45	6.02
Leaf 5	1582	6620.43	5.18
Mean	1444.60	6554.90	5.18
SD	154.49	119.34	0.76

Table 2 FCS indices of cypselae sample for 4 populations.

Sample	Embryo			Endosperm		
	Events	Geometric	CV	Events	Geometric	CV
Guangzhou 1	2392	6145.12	9.23	322	12741.12	10.41
Guangzhou 2	2013	6494.26	12.22	260	13361.00	12.32
Guangzhou 3	1661	6506.35	9.52	231	12743.40	10.69
Guangzhou 4	2452	6433.06	9.23	233	12772.48	10.63
Guangzhou 5	2134	6826.26	10.55	376	13374.45	11.89
Qingyuan	4396	402.05	6.60	503	802.69	8.29
Qinzhou	3068	343.53	11.29	787	703.45	10.68
Maoming	4337	385.13	7.42	412	780.53	7.72
Mean	2806.63	4191.97	9.51	390.50	8409.89	10.33
SD	1043.95	3164.53	1.87	186.13	6338.06	1.59

Table 3 Peak indices for for 4 populations.

Sample	PI 1	PI2
Guangzhou 1	1.04	2.07
Guangzhou 2	1.03	2.06
Guangzhou 3	1.02	1.96
Guangzhou 4	1.00	1.99
Guangzhou 5	0.97	1.96
Qingyuan	/	2.00
Qinzhou	/	2.05
Maoming	/	2.03
Mean	1.01	2.02
STD	0.03	0.04

Note: The peak indices 1 (PI1) mean peak value of leaf compared to the mean peak of embryo. The peak indices 2 (PI2) mean peak value of embryo compared to the mean peak of endosperm.

Histograms of the flow cytometry of 4 populations of *Praxelis clematidea*.

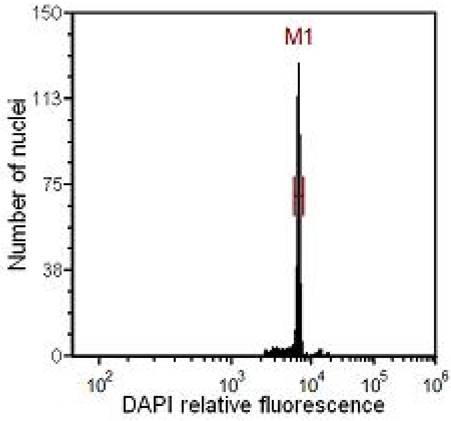

Guangzhou leaf1

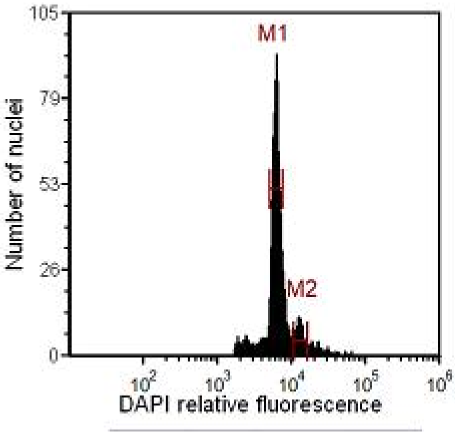

Guangzhou cypselae1

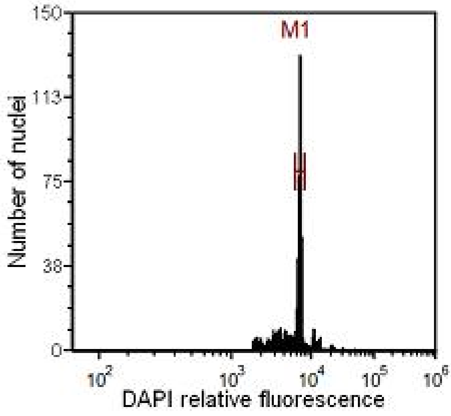

Guangzhou leaf 2

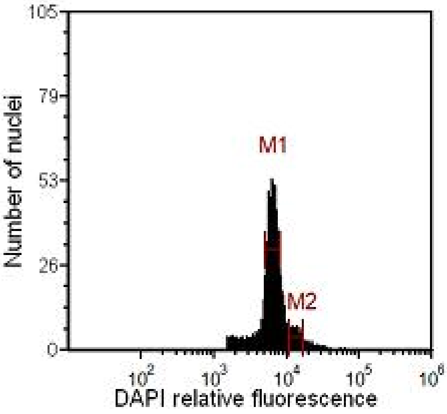

Guangzhou cypselae 2

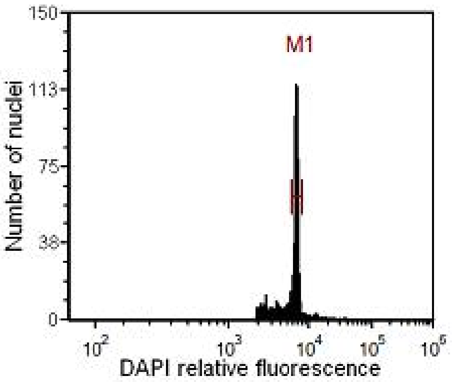

Guangzhou leaf 3

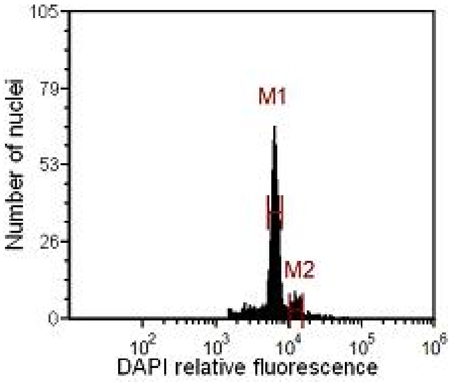

Guangzhou cypselae 3

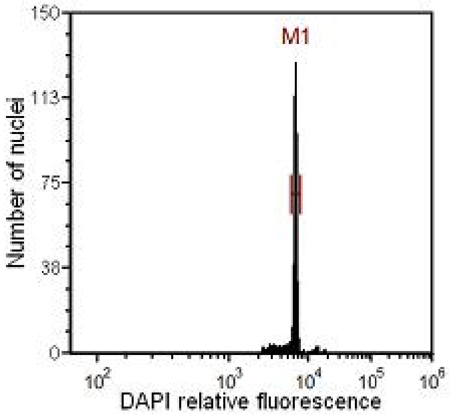

Guangzhou leaf 4

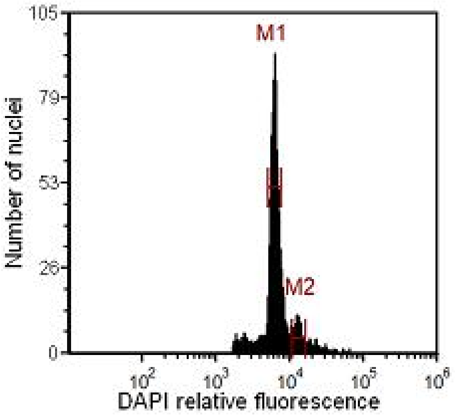

Guangzhou cypselae 4

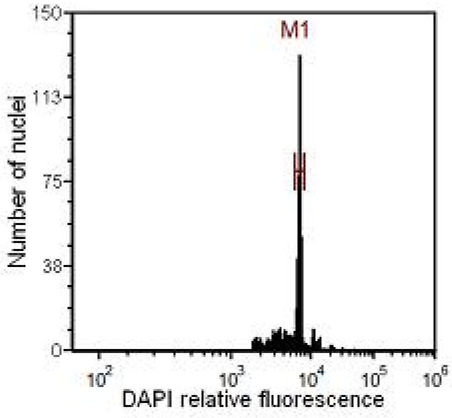

Guangzhou leaf 5

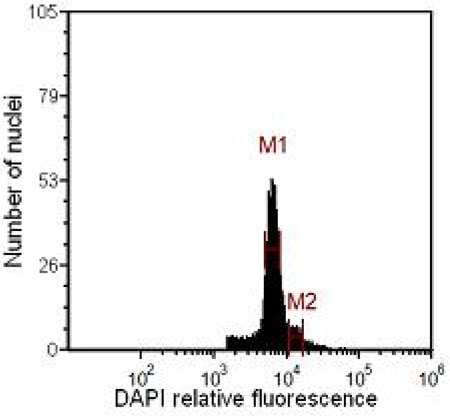

Guangzhou cypselae 5

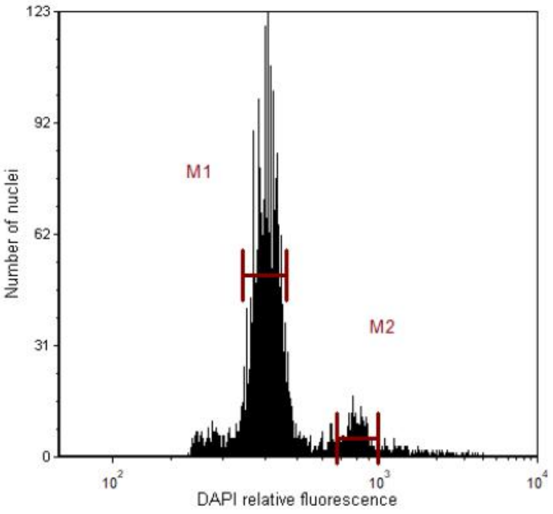

Qingyuan cypselae

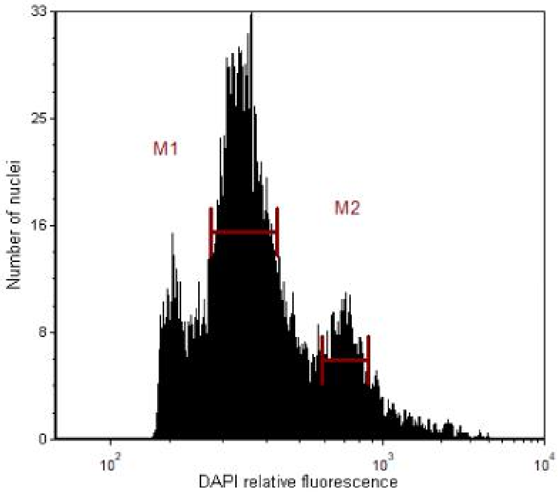

Qinzhou cypselae

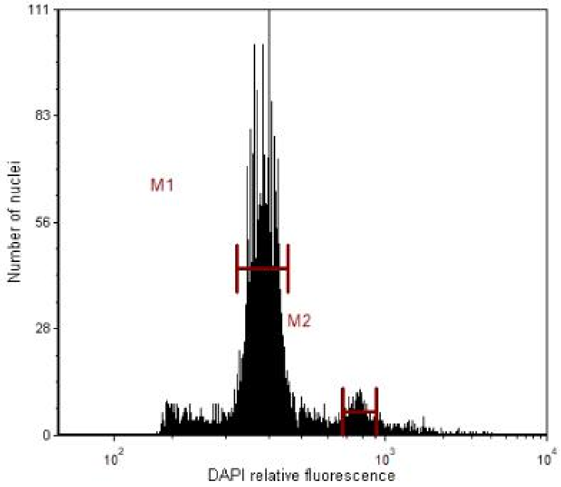

Maoming cypselae
